# Coronavirus Antibodies in Bat Biologists

**DOI:** 10.3201/eid1406.070964

**Published:** 2008-06

**Authors:** Lauren J. Stockman, Lia M. Haynes, Congrong Miao, Jennifer L. Harcourt, Charles E. Rupprecht, Thomas G. Ksiazek, Terri B. Hyde, Alicia M. Fry, Larry J. Anderson

**Affiliations:** * Centers for Disease Control and Prevention, Atlanta, Georgia, USA; † Atlanta Research and Education Foundation, Decatur, Georgia, USA; 1 These authors contributed equally to this article.

**Keywords:** coronavirus, mammologists, severe acute respiratory syndrome, letter

**To the Editor:** Severe acute respiratory syndrome–associated coronavirus (SARS-CoV) is a new coronavirus that caused an epidemic of 8,096 cases of SARS and 774 deaths during 2002–2003 (*1*). Attempts are ongoing to identify the natural reservoir of SARS-CoV. Several horseshoe bat species (*Rhinolopus* spp.) from Asia (*2*,*3*) and a sample of bats from Africa (*4*) have been found to be infected by and potential reservoirs for various SARS-like CoVs and various CoVs that are not SARS-like (*2*–*4*). However, transmission of bat SARS-CoV from bats to humans has not been reported.

During October 2005, we looked for serologic evidence of infection among bat biologists attending an international meeting in the United States. After giving informed consent, volunteer biologists completed an anonymous survey and provided 10 mL of blood. Serum samples were tested at the Centers for Disease Control and Prevention (CDC) for antibodies against inactivated human SARS-CoV and against recombinant, expressed SARS-CoV nucleocapsid protein (SARS-CoV N) by enzyme immunoassays (EIAs) as described (*5*,*6*). This study was approved by the CDC Institutional Review Board.

Of 350 registered biologists, 90 (26%) participated. Of participants, 89% had worked with or studied bats in North America, 21% in South America, 11% in Africa, 8% in Asia, 7% in Europe, and 6% in Australia. The primary genera studied by participants were *Myotis* (24%), *Tadarida* (13%), and *Eptesicus* (10%). A total of 20 (23%) participants had worked with or had contact with horseshoe bat species (*Rhinolopus* spp.). Because this genus has 69 species, distributed from Australia to Europe, some participants who indicated that they worked with the *Rhinolopus* spp. may likely have worked with species found outside of Asia. Involvement with bats most often consisted of capturing or handling them in the field (90%), followed by capturing or handling them in the laboratory (36%). Urine and feces were encountered most frequently (“always” or “most of the time” by 66%–68% of participants); contact with blood, saliva, or tissues and bites or scratches reportedly occurred less often (“always” or “most of the time” by 4%–28% of participants).

The serum samples from all 90 participants were negative for antibodies against inactivated SARS-CoV, and samples from all but 1 were negative for SARS-CoV N protein. The 1 positive sample gave a strong signal (optical density 1.08 at 405 nm at a 1:400 dilution) by SARS-CoV N protein EIA and against SARS-CoV N by Western blot but gave no reactivity against recombinant SARS-CoV spike protein or inactivated SARS-CoV by either EIA or Western blot. Because the N protein has a region that is relatively conserved among all known coronaviruses (*7*), the antibodies against SARS-CoV N protein could have been induced by other CoVs. Previous studies have demonstrated that SARS-CoV N protein can cross-react with polyclonal antiserum induced by group 1 animal CoVs (*8*).

To address the possibility that the antibodies from this serum sample were not specific to SARS-CoV, we tested it against recombinant N proteins of human CoVs, HCoV-229E, HCoV-OC43, NL63, and HKU-1. The serum reacted to all 4 N proteins, by EIA and Western blot, at titers of 400–1,600. We then tested the sample against 3 recombinant fragments of the N protein from each of 3 viruses: SARS-CoV, HCoV-229E, and HCoV-OC43. One of these fragments, N2, contains a highly conserved motif (FYYLGTGP) that should detect cross-reacting antibodies; the other 2 fragments should detect antibodies specific to the strain or group. The serum reacted to 2 of 3 fragments from HCoV-OC43 and -229E but to only the N2 fragment with the conserved motif from SARS-CoV (Figure), which suggests that the antibodies against SARS-CoV N were likely induced by a CoV that was not SARS-like.

**Figure:**
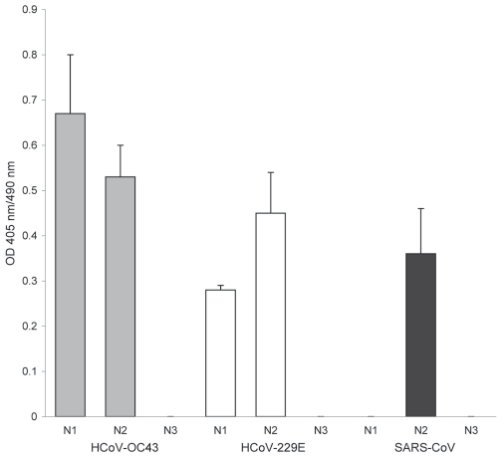
Antibody reactivity to coronavirus (CoV) nucleocapsid (N) protein fragments by ELISA. A set of recombinant protein fragments covering the N protein sequence of human CoV (HCoV)–OC43, HCoV-229E, and severe acute respiratory syndrome (SARS)–CoV were used as antigen; the serum (1:400 dilution) from the participant was tested by ELISA. The fragments include the following HCoVs: HCoV-OC43 N1 (aa 1–119), HCoV-OC43 N2 (aa 120–332), HCoV-OC43 N3 (aa 333–448), HCoV-229E N1 (aa 1–74), HCoV-229E N2 (aa 75–311), HCoV-229E N3 (aa 312–389), SARS-CoV N1 (aa 1–105), SARS-CoV N2 (aa 106–324), and SARS-CoV N3 (aa 325–422). The HCoV-OC43, HCoV-229E, and SARS-CoV fragments were coated at 4 × 10^–7^ M, 2.5 × 10^–3^ M, and 8 × 10^–8^ M, respectively. The N-terminal of the N protein contains a highly conserved motif (FYYLGTGP) found in all CoVs (*7*). This conserved motif is found in HCoV-OC43 N2, HCoV-229E N2, and SARS-CoV N2 recombinant protein fragments. The sizes of the expressed protein fragments used in this study were confirmed by sodium dodecyl sulfate–polyacrylamide gel electrophoresis. In addition, the reactivity of each protein fragment was confirmed by using Western blot with the anti-His antibody and the respective convalescent-phase serum. The mean optical density (OD) of absorbance at 405 nm (490-nm reference) of duplicate wells is shown. Error bars represent the standard deviation of duplicate wells.

If the antibodies were induced by a SARS-like CoV infection, we would expect to have also detected antibodies against recombinant S protein (*9*) or recombinant fragments representing antigenically distinct regions of the N protein of SARS-CoV. We did not detect either; instead, we detected antibodies against the antigenically distinct N fragments from group 1 and 2 human CoVs. Thus, this survey of a sample of bat biologists, who were exposed primarily to North American bats but also to bats from Asia and Africa, showed no evidence of SARS-like CoV infection.

Our survey found no evidence of SARS-CoV transmission from bats to humans. However, since the conclusion of this study, Dominguez et al. found coronavirus RNA in bats in North America, particularly *Eptesicus fuscus* and *Myotis occultus* (*10*), 2 species of the genera handled by 25% of the participants in our survey. Of interest is whether the bat biologists who worked with these bats might be at risk for infection with group 1 bat CoVs. Unfortunately, the high likelihood of infection with human group 1 CoVs will make it difficult to address this question. Additional studies of bat SARS-CoV infections in a larger number of persons who have been in contact with the species found to be positive for SARS-like CoV are needed before the risk for SARS-like CoV transmission from bats to humans can be clearly understood.
